# Pain Assessment and Its Effect on Pain Management During Emergency Medical Services—A Descriptive Study in the Tampere University Hospital Area of Finland

**DOI:** 10.1111/aas.70047

**Published:** 2025-05-07

**Authors:** Saara M. Lidauer, Sanna Hoppu, Kaius Kaartinen, Martin H. Lidauer, Maija‐Liisa Kalliomäki

**Affiliations:** ^1^ Faculty of Medicine and Health Technology, Tampere University Tampere Finland; ^2^ Emergency Medical Services, Centre for Prehospital Emergency Care, Pirkanmaa Wellbeing Services County Tampere Finland; ^3^ Natural Resources Institute Finland (Luke) Jokioinen Finland; ^4^ Department of Anaesthesiology and Pain Tampere University Hospital, Pirkanmaa Wellbeing Services County Tampere Finland

**Keywords:** acute pain, critical care, emergency medical services, pain assessment, pain evaluation, pain management

## Abstract

**Background:**

Pain management is an important part of prehospital care. Pain assessment and recognition are inseparable in adequate pain treatment. However, relatively scarce literature is available from Nordic and European countries. We investigated the current practices of pain assessment and management in the Emergency Medical Service (EMS) of one Finnish hospital area.

**Methods:**

The cohort data were originally collected prospectively to assess various quality processes in EMS. This study was designed as a descriptive, retrospective, register‐based cohort study, analysing data from 7245 patients encountered by EMS over a one‐month period in the Tampere University Hospital area of Finland in 2021. Pain levels were primarily assessed using the numeric rating scale (NRS). Records were combined with auxiliary data from the National Emergency Response Centre Agency. We grouped patients into six age groups and recorded dispatch categories in seven groups to clarify the impact of age and dispatch category on pain assessment and intensity. We used crosstabulation and Pearson's chi‐square test for statistical analyses; we also applied a linear mixed model to analyse the effect of pain medication on pain intensity.

**Results:**

Pain was assessed once in 2586 (36%) patients and then reassessed a second time for 707 (27%) of those patients. Age and dispatch category affected pain intensity (*p* < 0.001): Patients under 66 reported higher pain intensity than patients over 66 years. In dispatch categories, “oxygen deficiency” and “non‐mechanical accident or exposure,” reported pain intensity was minimal compared to other categories. Of the patients with a pain assessment, 611 (24%) experienced pain equal to or greater than 4 on the NRS scale. We found that assessment with a high NRS level was associated with a higher likelihood of administering potent pain medication.

**Conclusion:**

The prevalence of pain assessment is relatively low. The diverse nature of EMS interventions must be considered when improving pain management strategies.

## 1 Introduction

Reports indicate that 70% of prehospital trauma patients experience pain [1]. The Emergency Medical Services (EMS) are critical in early pain recognition, assessment, and management. Effective pain management in patient care is crucial for reducing patient suffering and improving outcomes. Receiving adequate pain relief is humane and a basic human right [2].

Besides being an uncomfortable sensation, pain is a sign of actual or potential tissue damage [3]. Psychological factors can significantly influence the perception of the pain experience [3, 4]. The EMS commonly assess pain by interviewing the patient and using various rating scales, most commonly the numeric rating scale (NRS) from 0 to 10, the visual analogue scale (VAS), and the verbal rating scale (VRS), which consists of a list of five adjectives to describe pain intensity. The choice of scale used does not significantly impact the outcome of pain assessment [5]. Pain is assessed by observing the physiological signs and behavior of patients who are unconscious or otherwise cannot communicate verbally. Pain medication is chosen based on the severity and type of pain and patient‐related factors, such as age or kidney function. However, deficiencies in pain assessment and management have been reported [6, 7]. Unlike in a hospital environment, the EMS nurse paramedics often independently administer treatment in a prehospital setting.

The systematic approach to pain measurement and documentation within the Finnish EMS remains unclear, and existing data are limited. This also holds true for other European EMS systems [8, 9]. Although pain management is crucial in EMS, this topic has not been extensively highlighted in the literature. A recent large multicenter study concludes that pain is often ignored and undertreated in EMS [10]. The primary focus of our study was on the prevalence of pain assessment within one Finnish EMS region. Additionally, we investigated whether the patient's age, dispatch category, sex, and level of consciousness influenced pain measurement, intensity, and the administration of analgesics and whether the analgesics administration affected the pain level.

## 2 Methods

### 2.1 Setting

Data collection occurred in the Tampere University Hospital area of Finland, covering 15,550 km^2^, with a population of 527,478 in 2021. The Emergency Medical Services (EMS) are organized by the Pirkanmaa Wellbeing County's Centre of Prehospital Emergency Care [11, 12, 13]. The EMS in this area consists of First Responding Units (FRU), 38 ambulances (28 available 24/7), an EMS field commander unit, and on‐call emergency physicians with access to a helicopter or ambulance [14]. The FRUs, which firefighters typically staff, provide first aid but do not transport patients. All ambulances in this area are Advanced Life support (ALS) units, each staffed by at least one nurse paramedic [15]. The National Emergency Response Centre Agency (ERC Agency) handles all emergency calls. Salminen et al. [16] provide a detailed analysis of the ERC Agency's operation, including the determination of dispatch categories and priorities within the Finnish EMS.

Pain medications administered on the scene or during transport include paracetamol, non‐steroidal anti‐inflammatory drugs (NSAIDs), esketamine, methoxyflurane, and strong opioids (e.g., oxycodone and fentanyl). According to EMS guidelines, an NRS pain score of 4 (or corresponding VAS or VRS value) prompts additional analgesics, specifically the strong opioids, esketamine, or methoxyflurane. Paracetamol and NSAIDs are options for pain treatment with an NRS pain value below 4. Pain management should begin within 15 min of patient contact, aiming to reduce the NRS pain score below 4 during transport. Transport occurs in an ambulance equipped with monitoring capabilities when opioids are administered. The EMS team should routinely reassess pain during transport if the NRS pain score is 4 or higher [17].

### 2.2 Study Design and Data Collection

The cohort data were originally collected prospectively to assess various quality processes in EMS. However, this study was designed as a descriptive, retrospective, register‐based cohort study. The study was conducted by analysing data collected from August 1, 2021 to August 31, 2021. The dataset includes records of various vital signs and symptoms from all patients who received prehospital care from the ALS units during EMS missions. Pain levels were primarily assessed using the NRS scale. The ALS teams recorded the collected information on patient‐specific paper templates or by directly uploading it to the electronic database at Pirkanmaa Wellbeing County's Centre of Prehospital Emergency Care. Notably, no changes were made to the emergency care procedures or protocols during the data collection period. The ALS teams were instructed on the importance of meticulously recording the required information.

Research assistants manually entered data from the EMS patient forms into the electronic database. This dataset included information on the patients' demographics (sex, age), medical history, potential disabilities, vital functions, pain assessments, and the medications and treatments administered. Additional data regarding dispatch priority and category, transportation codes, and priorities were extracted from the national ERC Agency system and added to the collected patient data. The combined data were also pseudonymised and used in other studies [16]. Authors KK and SH performed data validation, and erroneous entries were excluded. For this study, we classified the 61 recorded dispatch categories into seven groups (Table S1) by following the Finnish EMS guide's classifications [18]. From the edited data for this study, we calculated the average duration of the key activities during EMS missions: time to reach the patient, time on the scene, transport time to the hospital, and admission time at the hospital.

### 2.3 Statistical Analyses

#### 2.3.1 Prevalence of Pain Assessment, Pain Intensity, and Medication

The prevalence of pain assessment was analysed by the presence of pain scores in patient records. We categorized pain intensity into five classes corresponding to the VRS: no pain (NRS/VAS 0), mild pain (NRS/VAS 1–3), moderate pain (NRS/VAS 4–6), severe pain (NRS/VAS 7–9), and intolerable pain (NRS/VAS 10). We classified the Glasgow coma scale (GCS) observations into three categories: severe (GCS 3–8), moderate (GCS 9–12), and mild or no (GCS 13–15) disturbance in level of consciousness. Data analysis was conducted using crosstabulation and Pearson's chi‐squared‐test. The statistical significance level was considered at *p* < 0.05.

#### 2.3.2 Effect of Medication on Pain Level

We excluded all patients for this analysis who had no pain in their first pain assessment or received no pain medication; thus, our dataset consisted of 1262 pain records from 905 patients, of whom 357 (39%) patients also had a record of a second pain assessment. We analyzed the pain intensity observations to assess whether an association existed between the type of medication and the pain intensity and how large an effect the medication had on pain intensity by using the following linear mixed model:

$$y_{\mathrm{ijk}}=\mu +m_{\mathrm{ij}}+p_{k}+e_{\mathrm{ijk}}$$

where $y_{\mathrm{ijk}}$ is a pain intensity observation that was made at assessment time *j* on patient *k*, who received after the first assessment a treatment that belonged to pain medication group *i*; μ is the general mean of the pain intensity observations; $m_{\mathrm{ij}}$ is the fixed effect interaction of medication group *i* × assessment time *j*, where medication groups were nine (paracetamol, NSAIDs, opioid, esketamine, methoxyflurane, combination 1 with NSAIDs and paracetamol, combination 2 with NSAIDs and/or paracetamol and opioid, esketamine, or methoxyflurane, combination 3 with two or more of the following: opioid, esketamine, and methoxyflurane, and a group for no pain medication), and assessment times were two (1st assessment, 2nd assessment); $p_{k}$ is the random effect of patient *k*; and $e_{\mathrm{ijk}}$ is the random residual effect. We assumed the model's random effects were normally distributed with $p∼N\left(O,I\sigma_{p}^{2}\right)$ and $e∼N\left(O,I\sigma_{e}^{2}\right)$, where $\sigma_{p}^{2}$ is the variance of the patient effect and $\sigma_{e}^{2}$ is the variance of the residual effects. During the model development, we assessed the significance of the fixed effects by fitting a least square model that included the effects of age, sex, and interaction between the medication group × assessment time. The patient's age and sex did not significantly affect the pain intensity level; therefore, these effects were not included in the model. To calculate statistical significance levels, we applied the Bonferroni adjustment to account for multiple pairwise comparisons.

We used IBM SPSS Statistics software version 29.0.1.0 for Windows to perform the statistical analyses.

### 2.4 Ethics

The Tampere University Hospital research director approved (no. R21641) this retrospective register‐based cohort study. Finnish law states that patient consent and an ethics committee statement are not required if no patient interventions exist.

## 3 Results

### 3.1 Characteristics of Emergency Medical Service Intervention

The EMS recorded 7245 missions during the data collection period from August1, 2021 to August 31, 2021. The time between the call to the ERC Agency and when the EMS unit met the patient took an average of 12.2 min. The time spent on the scene was an average of 20.6 min, and transport to the hospital took an average of 19.7 min. The total time from the call until the patient was taken into hospital care averaged 68.2 min. The median and mean of the transportation time were 14.0 and 19.7 min, respectively. The lengthiest transportation took 136.0 min.

We included in the analyses all patients who received prehospital care from EMS. The patients' ages ranged from 0 to 103, with an almost equal distribution of both sexes. Table 1 presents the demography and patient characteristics of the recorded data. The most common reason for EMS intervention was a disturbance in vital functions, accounting for 1971 (27%) patients of all recorded EMS cases. Among these 1971, 675 (34%) presented with chest pain, and 445 (23%) experienced breathing difficulty. Most pain registrations were for patients with mild or no disturbances in consciousness. GCS scores were ≤ 8 for 11 (0.4%) out of 2570 patients with pain and GCS scores assessed.

**TABLE 1:** Descriptive statistics of the patient data recorded by the Emergency Medical Service (EMS).

Characteristics [unit]	Patients met by EMS (*N* = 7245)		Pain assessed patients (*N* = 2586)	
	*N*	% or Mean (± SD)	*N*	% or Mean (± SD)
Age [years]	6287	60.1 (± 24.9)	2579	62.2 (± 23.3)
Sex [% female]	6283	51.5	2574	51.4
Dispatch category	7218		2584	
Disturbance of vital functions [%]	1971	27.3	803	31.1
Other illness, births, bleeding without injury [%]	1793	24.8	671	25.9
Injury, violence or a mechanical accident [%]	1516	21.0	505	19.5
Blunt injury [%]	930	61.3	389	77.0
Sharp injury [%]	77	5.1	25	5.0
Isolated brain injury [%]	87	5.7	36	7.1
Multiple disability [%]	35	2.3	10	2.0
Pain as the main symptom [%]	1225	17.0	389	15.1
Non‐mechanical accident or exposure [%]	367	5.1	74	2.9
Unspecified symptom or reason for needing help [%]	323	4.5	134	5.2
Oxygen deficiency [%]	23	0.3	8	0.3
Glasgow coma scale [3–15]	6040	14.7 (± 1.5)	2570	14.9 (± 0.7)

### 3.2 Pain Assessment and Pain Intensity Across Age Groups and Dispatch Categories

**FIGURE 1:**
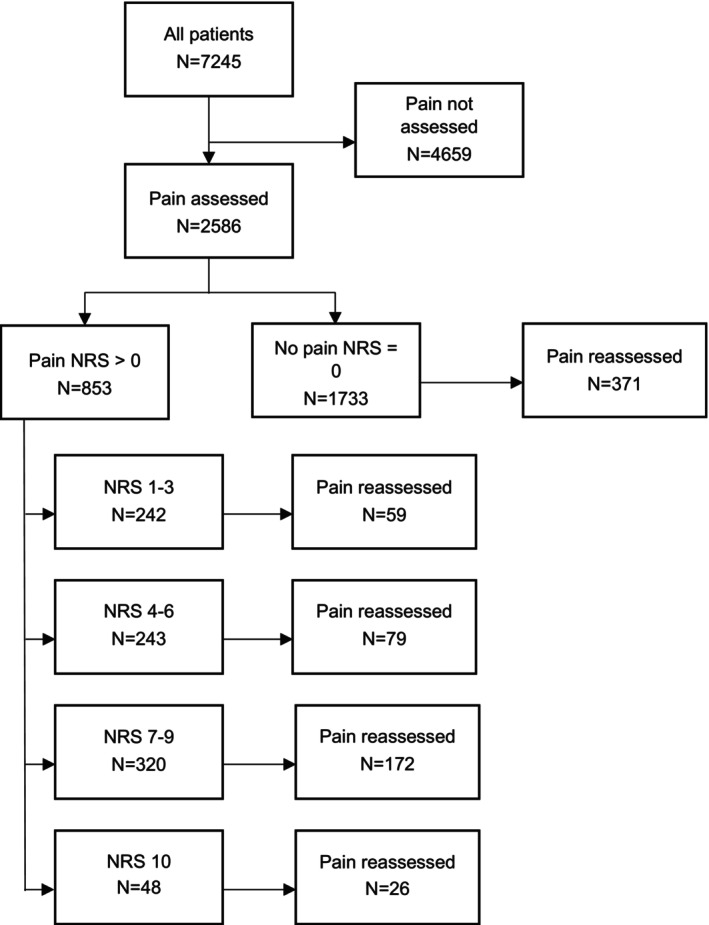
Prevalence of pain assessment and its intensity measured with numeric rating scale (NRS). NRS 1–3 = *mild pain*, NRS 4–6 = *moderate pain*, NRS 7–9 = *severe pain*, NRS 10 = *intolerable pain*.

The ALS teams assessed pain at least once in 2586 (36%) patient cases; reassessment was performed in 707 (27%) patients. Among patients assessed for pain, 1733 (67%) experienced no pain, 242 (9%) mild pain, 243 (10%) moderate pain, 320 (12%) severe pain, and 48 (2%) patients experienced intolerable pain (Figure 1). Altogether, 611 (24%) patients reported pain equal to or greater than 4 on the NRS. The prevalence of pain assessments varied across age groups (Table 2) and dispatch categories (Table 3) for the initial assessment and reassessment (*p* < 0.001). We found no difference in pain intensity between men and women.

**TABLE 2:** Effect of age on pain prevalence and intensity.

Age [years]	0–16	17–35	36–50	51–65	66–80	≥ 81	Total
	*N*	*N*	*N*	*N*	*N*	*N*	*N* [%]
Total (patients/age group)	312	1010	756	935	1648	1626	6287
First assessment*							
No pain (NRS 0)	59	201	169	257	534	507	1727 (67.0)
Mild pain (NRS 1–3)	8	41	38	34	60	61	242 (9.4)
Moderate pain (NRS 4–6)	10	56	26	42	60	48	242 (9.4)
Severe pain (NRS 7–9)	4	59	49	66	99	43	320 (12.4)
Intolerable pain (NRS 10)	5	7	13	8	8	7	48 (1.8)
Total *N* [%]	86 (3.3)	364 (14.1)	295 (11.5)	407 (15.8)	761 (29.5)	666 (25.8)	2579 (100)
Assessed/age group [%]	27.6	36.0	39.0	43.5	46.2	41.0	
Reassessment**							
No pain (NRS 0)	7	34	31	58	146	121	397 (56.2)
Mild pain (NRS 1–3)	3	17	16	27	31	23	117 (16.5)
Moderate pain (NRS 4–6)	7	29	16	27	32	22	133 (18.8)
Severe pain (NRS 7–9)	2	5	14	12	15	7	55 (7.8)
Intolerable pain (NRS 10)	1	1	3	0	0	0	5 (0.7)
Total *N* [%]	20 (2.8)	86 (12.2)	80 (11.3)	124 (17.5)	224 (31.7)	173 (24.5)	707 (100)
Reassessed/1st assessment [%]	23.3	23.6	27.1	30.5	29.4	26.0	

* Pain intensity was significantly associated with age, $\chi^{2}$ (20, 2579) = 108.8, *p* < 0.001.

** Pain intensity was significantly associated with age, $\chi^{2}$ (20, 707) = 81.1, *p* < 0.001.

**TABLE 3:** Effect of dispatch category on pain prevalence and intensity.

Dispatch category	Disturbance of vital functions	Oxygen deficiency	Injury, violence, or a mechanical accident	Non‐mechanical accident or exposure	Other illness, births, bleeding without injury	Pain as the main symptom	Unspecified symptom or reason for needing help	Total
	*N*	*N*	*N*	*N*	*N*	*N*	*N*	*N* [%]
Total (patients/dispatch category)	1971	23	1516	367	1793	1225	323	7218
First assessment*								
No pain (NRS 0)	584	8	284	69	576	122	88	1731 (67.0)
Mild pain (NRS 1–3)	64	0	81	2	45	42	8	242 (9.4)
Moderate pain (NRS 4–6)	68	0	65	1	21	71	17	243 (9.4)
Severe pain (NRS 7–9)	83	0	66	1	25	128	17	320 (12.4)
Intolerable pain (NRS 10)	4	0	9	1	4	26	4	48 (1.8)
Total *N* [%]	803 (31.1)	8 (0.3)	505 (19.5)	74 (2.9)	671 (26.0)	389 (15.0)	134 (5.2)	2584 (100)
Assessed/dispatch category [%]	40.7	34.8	33.3	20.2	37.4	31.8	41.5	
Reassessment**								
No pain (NRS 0)	165	0	44	10	116	18	42	395 (56.0)
Mild pain (NRS 1–3)	39	0	32	0	15	21	10	117 (16.6)
Moderate pain (NRS 4–6)	32	0	33	1	7	43	17	133 (18.9)
Severe pain (NRS 7–9)	15	0	10	0	4	21	5	55 (7.8)
Intolerable pain (NRS 10)	0	0	1	0	0	4	0	5 (0.7)
Total *N* [%]	251 (35.6)	0 (0.0)	120 (17.0)	11 (1.6)	142 (20.1)	107 (15.2)	74 (10.5)	705 (100)
Reassessed/1st assessment [%]	31.3	0.0	23.8	14.9	21.2	27.5	55.2	

* Pain intensity was significantly associated with dispatch category, $\chi^{2}$ (24, 2584) = 492.5, *p* < 0.001.

** Pain intensity was significantly associated with dispatch category, $\chi^{2}$ (20, 705) = 171.1, *p* < 0.001.

Most patients assessed had a disturbance in vital functions. However, pain assessments were conducted most frequently when the EMS intervention was unspecified (Table 3). Pain was likelier to be reassessed when the initial pain level was higher (Figure 1). Notably, pain was never assessed more than twice per patient. Age influenced pain intensity (*p* < 0.001). Patients over 66 years reported lower pain more often than younger patients. Mild pain was most common in the 36–50 age group, moderate pain among those aged 17–35, severe pain among those aged 51–65, and intolerable pain among those 0–16 and 36–50 (Table 2). Variations in pain reassessment also occurred across age groups.

Pain intensity varied across dispatch categories (*p* < 0.001). Patients with oxygen deficiency reported no pain, while those categorized under “non‐mechanical accident or exposure” reported minimal pain. Mild pain was predominantly experienced by patients grouped into the “injury, violence or a mechanical accident” category. Moderate, severe, and intolerable pain occurred most in patients grouped into the “pain as the main symptom” category (Table 3).

Among the 853 (33%) patients who reported pain (NRS > 0), 336 (39%) of the cases underwent a reassessment. Of these cases, 83 (25%) patients had unchanged pain, 242 (72%) experienced decreased pain intensity, and 11 (3%) reported increased pain.

### 3.3 Administration of Analgesics and Its Connection With Pain Assessment

Altogether, 669 (9%) patients received pain medication. Of these, 555 (83%) patients received a single type of medication: 216 (39%) received paracetamol, 49 (9%) NSAIDs, 273 (49%) opioids, 9 (2%) esketamine, and 8 (1%) methoxyflurane. Additionally, 114 (17%) patients received two or more medications. The most common combination was paracetamol and NSAIDs in 51 patients (45%) of all combinations. Altogether, paracetamol was administered to 285 (4%) patients, NSAIDs to 102 (1%), opioids to 338 (5%), esketamine to 22 (0.3%), and methoxyflurane to 13 (0.2%) from all 7245 patients. Pain was assessed in 383 (57%) patients who received pain medication and in 254 (72%) of those 353 patients who received opioids, methoxyflurane, esketamine, or a combination with these. Out of the 353 patients who received more potent medication, pain was reassessed in 182 (52%) patients.

### 3.4 Effect of Pain Medication on Pain Intensity

Out of all patients included in the analysis, the observed average pain level on the NRS scale was 5.2 in the first assessment and 3.4 in the reassessment. The overall mean of the pain intensity from both assessments was 4.8 (SD ± 2.9), yielding a coefficient of variation of 61%. The highest reduction in pain was found for patients medicated with NSAIDs and/or paracetamol and opioid, esketamine, or methoxyflurane (combination group 2), with a pain reduction from 8.7 to 5.8 (Table 4). All pain medications reduced the pain level.

**TABLE 4:** Estimated effect (± SE) of medication on pain level [by numeric rating scale, NRS] for different medication groups.

Medication group	Observations		Estimates		Difference 2nd–1st assessment estimates	
	1. assessment	2. assessment	1. assessment	2. assessment	Difference	Significance
Paracetamol	85	25	2.82 ± 0.28	1.85 ± 0.46	−0.97	ns
NSAID	20	6	5.30 ± 0.57	4.04 ± 0.94	−1.26	ns
Opioids	194	142	6.83 ± 0.18	4.45 ± 0.21	−2.38	***
Esketamine	2	2	8.50 ± 1.81	3.50 ± 1.81	−5.00	ns
Methoxyflurane	4	3	7.50 ± 1.28	3.75 ± 1.43	−3.75	ns
Combination 1^a^	24	6	4.92 ± 0.52	3.69 ± 0.93	−1.23	ns
Combination 2	19	10	8.68 ± 0.59	5.75 ± 0.76	−2.93	*
Combination 3	35	25	7.86 ± 0.43	5.15 ± 0.49	−2.71	***
No pain medication	522	138	4.63 ± 0.11	2.79 ± 0.20	−1.84	***

*Note:* ns, Estimate for 1st and 2nd assessments do not significantly differ.

^a^ Combination 1: NSAIDs and paracetamol; combination 2: NSAIDs and/or paracetamol and opioid, esketamine, or methoxyflurane; combination 3: two or more of the following: opioid, esketamine, and methoxyflurane.

* Estimate for 1st and 2nd assessments differs significantly at *p* < 0.05.

*** Estimate for 1st and 2nd assessments differ significantly at *p* < 0.001.

Table 4 summarizes the association between pain intensity and medication administered, as well as medication's effect on pain intensity. The estimated intraclass correlation coefficient was 0.51, meaning 51% of the observed variance in the pain level was explained by the patient effect, which is also the repeatability of the pain assessment observation between the first and second assessments.

## 4 Discussion

The present study examined the prevalence and management of pain in patients receiving prehospital emergency care from the Emergency Medical Services (EMS) in the Tampere University Hospital area of Finland. Pain was assessed in 2586 (36%) of all patients, with reassessment occurring in 707 (27%) of these cases. Factors such as age and dispatch category influenced the initial pain assessment. Pain was assessed less frequently in younger age groups than in older ones. Assessments were most common when the EMS intervention was related to an unspecified reason or for disturbance in vital functions. Overall, 853 (33%) patients reported experiencing pain during the initial assessment, with 611 (24%) reporting a pain level of 4 or higher on the numeric rating scale (NRS). Our findings highlight significant variability in pain assessment across age groups and medical conditions. This study provides an analysis of how EMS in a university hospital region assesses, analyzes, manages, and records pain in EMS patients, underscoring the importance of systematic pain assessment in emergency settings.

Törmä et al. [19] clarified pain management practices in a register‐based study in the Helsinki area, finding pain assessment in only 27 of 126 patients with recorded VAS/NRS values, with the most complete records for chest pain cases. Friesgaard et al.'s [20] study in Denmark found moderate to severe pain in 27.7% of EMS patients, mild or no pain in 40.1%, and unknown pain status in 32.2%. Compared to our study, they assessed pain in 67.8%. A large, recently published two‐year cohort study in Sweden revealed significant deficiencies in recorded pain assessment and management within the EMS. Only 23% of patients had their pain assessed using a validated scale, and 28% received analgesics despite pain‐related conditions being common reasons for contacting EMS [8].

Pain assessment occurred less frequently in younger age groups, yet these patients reported higher pain intensity than older individuals. Additionally, pain intensity and assessment frequency varied across dispatch categories, even among patients within the same category. These findings suggest that pain assessment is not uniformly conducted across all patient groups, potentially highlighting challenges in assessing pain in specific populations, such as children or those with unclear symptoms. It has to be noted that in the category “Pain as the main symptom,” only 389 (32%) patients were assessed at least once, and 107 (28%) were reassessed. The reasons underlying such a low percentage are unclear but might include urgency of stabilising other vital functions or managing trauma. The low percentage may also have been influenced by paramedics' judgment that pain assessment was unnecessary because patients were not experiencing any pain. Recording pain intensity is not mandatory, which might be another reason for the low assessment frequency. However, we found an intraclass correlation of 0.51 between the first and second pain assessments, indicating high‐quality assessment. A poor quality would result in a low intraclass correlation. Nevertheless, it should be noted that the reduction of pain level due to medication reduces the intraclass correlation.

Pain was assessed in 383 (57%) patients who received pain medication. Opioids, methoxyflurane, esketamine, or a combination of these were administered to patients with higher NRS levels. Notably, the first assessment occurred for 254 (72%) and reassessment for only 182 (52%) patients. However, for these patients, the frequency of the initial pain assessment was twice as high compared to the overall pain assessment frequency of 36%. Danish emergency medical technicians must reassess pain using the NRS after administering intravenous fentanyl [20], while Finnish EMS guidelines recommend monitoring without mandating a standardized method. However, the relatively low rate of reassessment suggests a potential gap in continuous pain management, which could adversely affect patient outcomes.

The results are generalisable to countries with EMS and healthcare systems like Finland's, particularly in other Nordic countries, despite some variations in practice. The population density is also similar in Finland, Norway, and Sweden, making the results easier to compare because the durations of the transports are alike. The Pirkanmaa area consists of the city of Tampere and the surrounding rural area, which has a lower population density. Consequently, the largest part of the transport was short city area transportation; this explains a skewed distribution of transporting time with the median and mean durations of the transport time of 14.0 and 19.7 min, respectively. The relatively short duration of transport can also be why the pain reassessment occurred only in 707 (27%) of the patients.

The experience and expression of pain can differ across cultures, suggesting that our results may apply to populations with characteristics akin to those in Finland [21, 22]. Our study shows a lack of systematic pain assessment. Similar challenges have been identified in the Nordic and some other European countries. Our study supports findings reported in these studies more recently [8, 9].

Our study has limitations. Due to its retrospective nature, we lacked comprehensive information on all patient characteristics data (e.g., age, sex, dispatch category, GCS), leading to heterogeneous data. Pain assessment timing relative to medication administration was unclear. The varying sizes of age groups, dispatch categories, and medication groups further complicated our analyses. Consequently, we cannot draw significant conclusions about pain medication usage because the number of patients who received analgesics was relatively small, and our data were collected over 1 month.

A key strength of our study was that the EMS practices remained unchanged during the data collection period, allowing for an authentic examination of their operations. The data were based on EMS staff records during routine practice, providing a realistic representation of patient management. Furthermore, we did not exclude patients based on age or dispatch category, ensuring a comprehensive analysis of EMS cases.

Adequate pain management is a fundamental patient right. These results indicate that pain assessment is inconsistent across patient groups, which may reflect challenges in specific populations, such as children or those with unclear symptoms. Acute pain management strategies are generally effective; nevertheless, more rigorous pain monitoring and treatment adjustments are needed, particularly for younger patients and those initially presenting with severe pain. The significant variation in pain intensity across patient demographics highlights the importance of context‐specific pain management strategies in prehospital care. In addition to this, the pain patient‐ and situation‐specific analgesic use is an interest of further research. Additionally, further research into the benefits and challenges of methoxyflurane in the Finnish EMS setting would be valuable.

## 5 Conclusions

This study underscores the complexity of pain management in prehospital care, with significant variations in pain prevalence, intensity, and treatment across different patient demographics and medical conditions. The findings suggest a need for more consistent pain assessment and reassessment practices, particularly in younger patients and those presenting with severe pain. Furthermore, the results highlight the importance of customized pain management strategies that consider the specific needs of different patient groups to improve pain outcomes in the prehospital setting.

## Author Contribution

M.‐L.K., S.H., and K.K. developed the study design. S.M.L. conducted the analyses and wrote the first draft. M.H.L. contributed to the statistical analyses. All authors contributed to the writing and approved the final manuscript.

## Conflicts of Interest

The authors declare no conflicts of interest.

## Supporting information


**Table S1.** Grouping of dispatch category: code and explanation.

## Data Availability

Research data are not shared.
