# Supplementary material for: Pain Assessment and Its Effect on Pain Management During Emergency Medical Services—A Descriptive Study in the Tampere University Hospital Area of Finland
Source: Acta Anaesthesiol Scand. 2025 May 7;69(6):e70047. doi: 10.1111/aas.70047 (PMC12056686; doi:10.1111/aas.70047)
Supplement: Supplementary file 1 — Table S1. Grouping of dispatch category: code and explanation. [file AAS-69-0-s001.docx]

SUPPLEMENTARY TABLE 1 Grouping of dispatch category: code and explanation

| **Disturbance of vital functions** | 485 Terrain rescue |
| --- | --- |
| 700 Lifeless | 487 Person rescue from above or below |
| 701 Resuscitation | 751Gas poisoning |
| 702 Loss of consciousness | 752 Poisoning |
| 703 Breathing difficulty | 753 Electric shock |
| 704 Chest pain | 754 Burn injury |
| 705 Rhythm disorder | 755 Burn injury, heat stroke |
| 706 Stroke | 756 Hypothermia |
| **Oxygen deficiency** | **Other illness, births, bleeding without injury** |
| 711 Airway obstruction | 761 Haemorrhage: from the mouth |
| 713 Hanging, strangulation | 762 Haemorrhage: gynecological/urological |
| 714 Drowning | 763 Haemorrhage: ear/nose |
| **Injury, violence or a mechanical accident** | 764 Leg ulcer/other |
| 741 Fall | 770 Attack of illness |
| 744 Wound | 771 Blood glucose imbalance |
| 745 Downfall | 772 Convulsion |
| 765 Impact | 773 Hypersensitivity reaction |
| 031 Gunshot | 774 General weakness |
| 032 Stab | 775 Vomiting, diarrhoea |
| 033 Beating, kicking | 791 Childbirth |
| 200 Road traffic accident: other | **Pain as the main symptom** |
| 202 Road traffic accident: small | 781 Abdominal pain |
| 203 Road traffic accident: moderate | 782 Headache/neck pain |
| 212 Rail traffic accident: small | 783 Back pain/hip pain |
| 213 Rail traffic accident: moderate | 784 Limb pain |
| 222 Water traffic accident: moderate | 785 Mental health issue |
| 235 Risk of an air traffic accident: moderate | 786 Body pain |
| 271 Off-road accident | **Unspecified symptom or reason for needing help** |
| 594 Tactical EMS | 707 Care facility transfer belonging to the EMS |
| **Non-mechanical accident or exposure** | 790 Alarm during call |
| 401 Building fire: small | 792 Standby, standby transfer |
| 402 Building fire: moderate | 793 Care facility transfer |
| 403 Building fire: large | 794 Other EMS transport/appointment task |
| 461 Prevention of damage: small | 862 Mutual administrative support for social work |
